# Genome-Wide Identification and Expression Pattern of the *NAC* Gene Family in *Panax notoginseng*

**DOI:** 10.3390/genes16030320

**Published:** 2025-03-07

**Authors:** Baihui Jin, Xiaolong Hu, Na Li, Xiaohua Li, Zebin Chen, Xinyu Zhao, Xiaoni Wu

**Affiliations:** 1 School of Agronomy and Life Sciences, Kunming University, Kunming 650201, China; jbhxkyz521@kmu.edu.cn (B.J.); shin777@163.com (X.H.); zbchenkmu@163.com (Z.C.); 18042797555@163.com (X.Z.); 2 Pu’er Agricultural Science Research Institute, Pu’er 665000, China; lina6550@126.com (N.L.); lxh9617_1@126.com (X.L.); 3 Faculty of Agronomy and Life Science, Kunming University, Kunming 650201, China

**Keywords:** *Panax notoginseng*, *NAC* gene family, *Alternaria panax*, expression pattern

## Abstract

Background: The NAC transcription factor family of genes is one of the largest families of transcription factors in plants, playing important functions in plant growth and development, response to adversity stress, disease resistance, and hormone signaling. In this study, we identified the number of members of the *Panax notoginseng* NAC (*PnNAC*) gene family and conducted a comprehensive analysis of their physicochemical characteristics, chromosomal location, evolutionary features, and expression patterns both in different parts of the plant at different growth stages and in response to infection by *Alternaria panax*. Methods: The *NAC* gene family in *P. notoginseng* was identified using Hidden Markov Model (HMMER) and National Center of Biotechnology Information Conserved Domain Database (NCBI CDD), and their physicochemical properties were analyzed with Perl scripts. Phylogenetic relationships were determined using Clustal Omega and FastTree, and gene structures were visualized with an R script. Promoter regions were analyzed with PlantCARE, motifs with MEME and ggmotif, and transcriptome data were processed using Hical Indexing for Spliced Alignment of Transcripts (HISAT2) and HTseq. Results: This study identified 98 PnNAC genes in *P. notoginseng*, analyzed their characteristics (protein lengths 104–882 aa, molecular weights 11.78–100.20 kDa, isoelectric points 4.12–9.75), location (unevenly distributed on 12 chromosomes, no tandem repeats), evolution, and expression patterns (distinct in different parts, growth stages, and after *A. panax* infection). Conclusions: *PnNAC* plays an important role in the growth and development of *P. notoginseng* and in its response to *A. panax*. *PnNAC* could be a candidate gene for further research on and functional analysis of *P. notoginseng* disease resistance.

## 1. Introduction

*P. notoginseng* (Burk.) F.H. Chen, a valuable traditional Chinese medicinal herb belonging to the Araliaceae family, is primarily distributed in the Wenshan Prefecture of Yunnan Province and the Baise region of Guangxi [1,2]. With a long history of medicinal use in China, *P. notoginseng* is commonly employed for hemostasis, pain relief, and anti-swelling and can be formulated into various preparations, such as capsules, powders, and injections [3,4,5,6]. However, during its growth, *P. notoginseng* is subjected to various diseases and stresses [7,8,9,10,11], which significantly threaten its growth and result in substantial economic losses.

Numerous studies have indicated that NAC transcription factors are involved in regulating plant growth and development, stress responses, disease resistance, and hormone signaling [12,13]. NAC transcription factors act as “molecular switches” during plant growth and development. Evans et al. [14] discovered through gene knockout techniques that *GhNAC20* in cotton positively regulates leaf senescence, and overexpression of *GhNAC20* can effectively delay leaf aging. During growth, plants frequently encounter environmental stresses such as drought, high temperatures, and low temperatures, and NAC transcription factors play a crucial role in plant responses to these stresses. Mao et al. [15] utilized transgenic technology to demonstrate that the wheat NAC transcription factor *TaNAC67* positively regulates wheat’s responses to drought, low temperature, and high salinity stresses. In addition to abiotic stresses, plants also face numerous biotic stresses from pathogens such as bacteria, fungi, viruses, and nematodes, and the NAC transcription factor family has significant functions in plant responses to biotic stress. Chen et al. [16] found that the NAC transcription factor *HvNAC6* in barley is positively regulated by ABA, thereby affecting barley’s resistance to powdery mildew. Throughout all growth stages, plant hormones influence plant development, and NAC transcription factors can regulate this process by modulating plant hormones such as ABA or GA. Shen et al. [17] cloned the NAC transcription factor *OsNAC2* from rice and found that overexpression of *OsNAC2* reduces the resistance of rice plants to salt and drought stresses, resulting in decreased yield under drought conditions. This was attributed to *OsNAC2*’s ability to directly bind to the promoter of the protein kinase gene *OsSAPK1*, thereby inhibiting the expression of the ABA signaling pathway-related gene *LEA3*. Therefore, it is likely that the NAC transcription factor gene family in *P. notoginseng* also plays an essential role in its growth and stress resistance.

Although the *NAC* gene family has been reported in various plants, including its closely related species *Panax ginseng* [18], there has been no report on the distribution, function, and structure of the NAC transcription factor gene family in *P. notoginseng*. Thus, this study employs bioinformatic analysis to comprehensively examine the number of NAC transcription factor gene family members, their physicochemical properties, chromosomal localization, evolutionary relationships, and their response patterns at different growth stages and during infection by the black spot disease pathogen. This research lays the foundation for further investigations into the functions of the *NAC* gene family in *P. notoginseng*.

## 2. Materials and Methods

### 2.1. Identification of the NAC Gene Family Members in P. notoginseng

The whole genome sequence, protein sequences, and annotation files of *P. notoginseng* were provided by Yang et al. [19]. The HMM file for the NAC transcription factor family (PF02365) was downloaded from Pfam (http://pfam.xfam.org/family/ accessed on 3 March 2025) [20]. The HMMER3 software [21] was utilized to align the protein sequences of *P. notoginseng* against the HMM file, with a threshold of 1 × 10^−5^ for screening the alignment results. Corresponding gene protein sequences were extracted, and sequences located outside chromosomes 1 to 12 were removed. After eliminating duplicates, the sequences were submitted to the NCBI CDD website (https://www.ncbi.nlm.nih.gov/cdd/ accessed on 3 March 2025) for protein structure prediction. Genes containing the NAC conserved domain were identified as members of the *P. notoginseng NAC* gene family. The gene identifiers were renamed according to their order on the chromosomes and their positions.

### 2.2. Prediction and Analysis of the Physicochemical Properties of P. notoginseng NAC Proteins

Using laboratory-developed Perl scripts, the length, molecular weight, and isoelectric point of the identified *NAC* gene family members in *P. notoginseng* were analyzed.

### 2.3. Phylogenetic Analysis of the P. notoginseng NAC Gene Family

To verify the phylogenetic relationships of the *NAC* gene family in *P. notoginseng*, protein sequences were subjected to multiple sequence alignments using Clustal Omega v1.2.2 [22]. Subsequently, a phylogenetic tree was constructed using the FastTree 2 software [23] with default parameters. The phylogenetic tree was visualized using the R package ggtree [24].

### 2.4. Gene Structure Analysis of the P. notoginseng NAC Gene Family

Annotation information for the *NAC* gene family members was extracted from the GFF3 annotation file of the *P. notoginseng* genome. The gene structure was visualized using a laboratory-developed R script.

### 2.5. Promoter Analysis of the P. notoginseng NAC Gene Family

Promoter regions (upstream 1500 bp) of the *NAC* gene family members were extracted from the whole genome sequence of *P. notoginseng*. The types, quantities, and functions of cis-acting elements were analyzed using the PlantCARE database (https://bioinformatics.psb.ugent.be/webtools/plantcare/html/ accessed on 3 March 2025) [25].

### 2.6. Motif Analysis of the P. notoginseng NAC Gene Family

Motif analysis of the *P. notoginseng NAC* gene family was conducted using the MEME software (https://meme-suite.org/meme/ accessed on 3 March 2025) [26] with the parameters: -nostatus -time 18,000 -maxsize 6,000,000 -mod anr -nmotifs 10 -minw 6 -maxw 100. Motifs were extracted and visualized using the R package ggmotif v.0.2.1 (https://cran.r-project.org/web/packages/ggmotif/index.html accessed on 3 March 2025).

### 2.7. Transcriptome Data Processing

Raw data for the expression analysis of the *P. notoginseng NAC* gene family were downloaded from the NCBI SRA database. The HISAT2 software v2.2.1 [27] was used to construct an index for the P. notoginseng genome, and then the transcriptome data were aligned to the reference genome. The aligned SAM format files were sorted into BAM files, and the HTseq software v2.0.3 [28] was utilized to extract the Counts values for each gene. Finally, a laboratory-developed R script was employed to convert the Counts values into FPKM values.

## 3. Results and Analysis

### 3.1. Identification of the P. notoginseng NAC Gene Family

**Figure 1:**
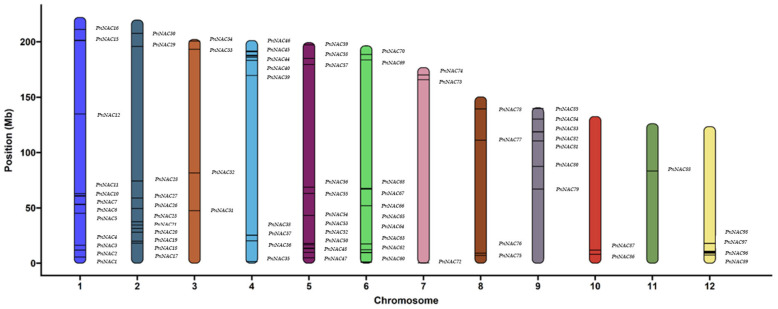
Chromosome mapping of *NAC* gene family members of *P. notoginseng*.

Based on the results from HMMER, a total of 98 *NAC* gene family members were identified in *P. notoginseng*. They were named *PnNAC1* to *PnNAC98* according to their relative positions on the chromosomes. These 98 genes were unevenly distributed across 12 chromosomes (Figure 1). Specifically, there are 16 *NAC* gene family members on chromosome 1, while only one member is found on chromosome 11. Most of these *NAC* gene family members are located at the ends of the chromosomes. The protein lengths of the 98 *NAC* gene family members range from 104 to 882 amino acids, with molecular weights varying from 11.78 to 100.20 kDa. Among them, *PnNAC91* has the longest protein length at 882 amino acids and a molecular weight of 100.20 kDa; *PnNAC44* and *PnNAC88* have the shortest protein lengths, both at 104 amino acids, with molecular weights of 11.78 kDa and 11.97 kDa, respectively. Of the 98 *NAC* gene family members, 42 genes have an isoelectric point greater than 7, while 56 genes have an isoelectric point less than 7. The range of isoelectric points is from 4.12 to 9.75 (Table 1).

### 3.2. Phylogenetic Analysis of the P. notoginseng NAC Gene Family

**Figure 2:**
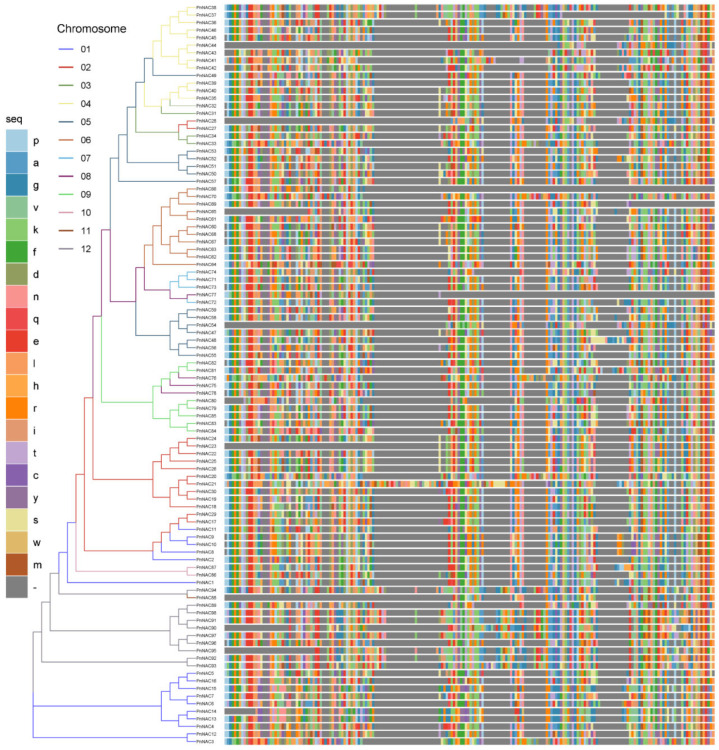
Phylogenetic tree of the *NAC* gene family of *P. notoginseng* and other species.

To better understand the phylogenetic relationships of the *NAC* gene family in *P. notoginseng*, the full-length protein sequences of the 98 *NAC* gene family members were aligned using the Clustal Omega software. A phylogenetic tree was constructed using FastTree (Figure 2), and the tree was visualized with the NAC domain sequences of each gene using ggtree. The *NAC* gene family in *P. notoginseng* is distributed across multiple subfamilies. Interestingly, there is a notable chromosomal preference observed within the *NAC* gene family, as members located on the same chromosome tend to exhibit higher similarity. For instance, *PnNAC49*, which belongs to the *NAC* gene family on chromosome 5, is more closely related to members of the *NAC* gene family on chromosome 4, with similar cases observed for *PnNAC77*, *PnNAC88*, and others. The 16 *NAC* gene family members on chromosome 1 are distributed across four different branches, with *PnNAC1* forming a distinct branch, suggesting that *PnNAC* may have undergone a unique domestication process during evolution.

Most *NAC* gene family members possess complete NAC domains; however, some genes exhibit varying degrees of large fragment deletions. Specifically, nine *NAC* gene family members—*PnNAC44*, *PnNAC25*, *PnNAC65*, *PnNAC54*, *PnNAC23*, *PnNAC88*, *PnNAC90*, *PnNAC95*, and *PnNAC14*—show certain fragment deletions at their N-termini, while *PnNAC66*, *PnNAC69*, and *PnNAC77* display large fragment deletions at their C-termini. These results indicate that the *NAC* gene family in *P. notoginseng* is diverse and exhibits chromosomal preference during evolution, and that some *NAC* genes have undergone significant fragment deletions throughout their evolutionary history.

### 3.3. Gene Structure Analysis of the P. notoginseng NAC Gene Family

**Figure 3:**
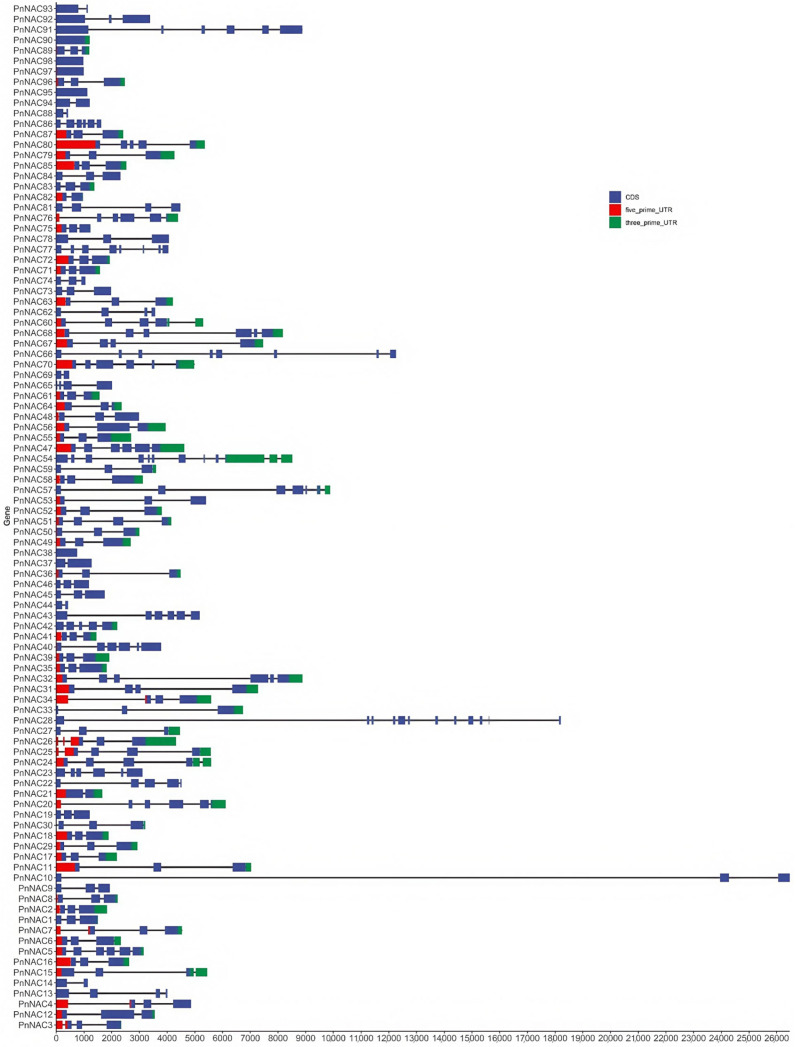
Structures of the *NAC* gene family members of *P. notoginseng*.

The structural analysis of the *NAC* gene family in *P. notoginseng* revealed that most members contain introns, with only a few genes lacking introns (Figure 3). Genes such as *PnNAC90*, *PnNAC95*, *PnNAC97*, *PnNAC98*, and *PnNAC38* do not have any introns. Among them, *PnNAC10*, which has the longest length, contains two introns, with the first intron measuring 2.38 kb.

### 3.4. Motif Analysis of the P. notoginseng NAC Gene Family

**Figure 4:**
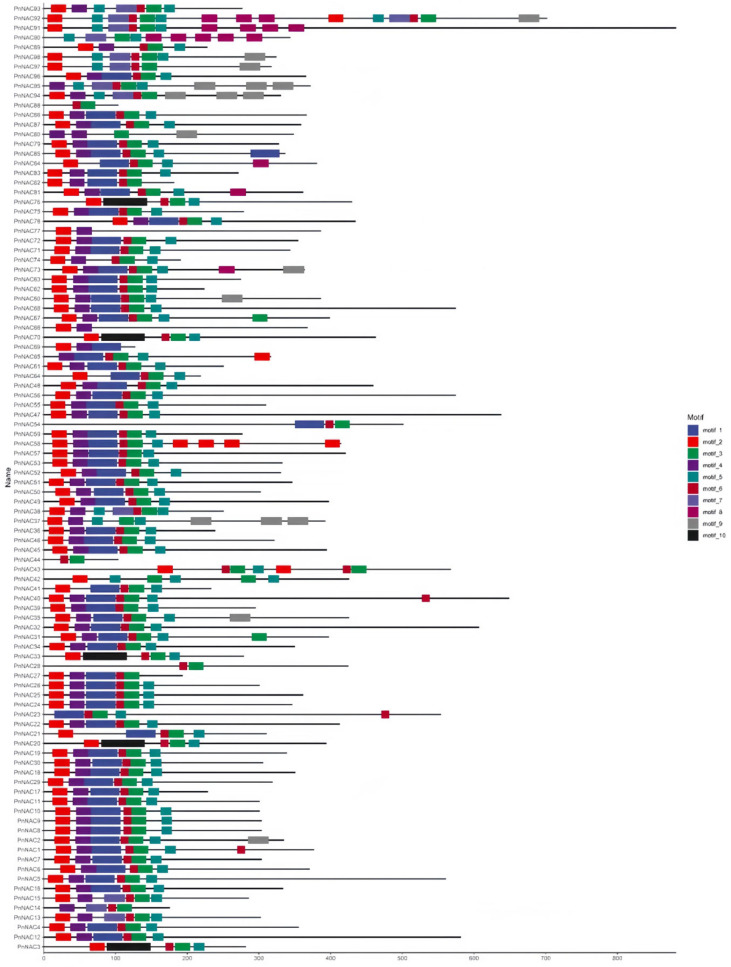
Motif analysis of the *NAC* gene family members of *P. notoginseng*.

**Figure 5:**
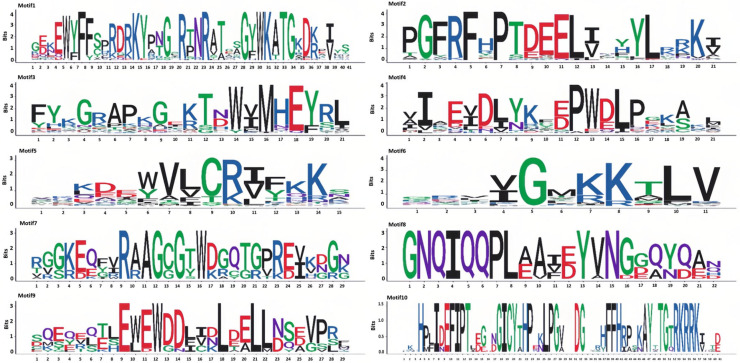
Gene structure and protein conserved domains of the *NAC* gene family members of *P. notoginseng*.

Motif analysis of the 98 members of the *NAC* gene family in *P. notoginseng* was conducted using the MEME software (Figure 4). A total of 10 distinct motifs were identified among the 98 *NAC* gene family members. The distribution of these 10 motifs is largely consistent across the NAC genes, with Motif 3 present in 95 of the *NAC* gene family genes. Notably, *PnNAC42*, *PnNAC43*, and *PnNAC92* each contain two instances of Motif 3. The position of Motif 1 is relatively fixed in most genes. Apart from Motif 10, the other motifs appear multiple times in certain genes. For instance, Motif 2 occurs twice in *PnNAC58*, and Motif 8 appears twice in *PnNAC90* (Figure 5).

### 3.5. Analysis of Cis-Acting Elements in the Promoters of the P. notoginseng NAC Gene Family

**Figure 6:**
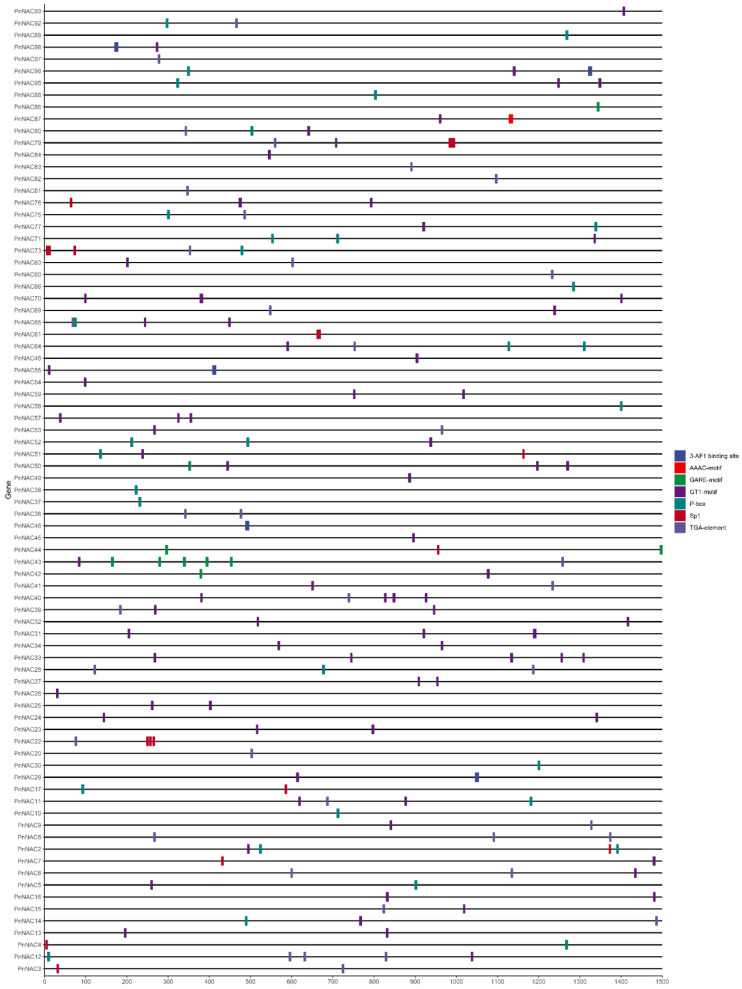
The cis-acting element of the *NAC* gene family of *P. notoginseng*.

To further investigate the functions of the *NAC* gene family in *P. notoginseng*, sequences 1500 bp upstream of the translation start sites of the NAC genes were extracted for cis-acting element analysis. A total of 107 cis-acting elements were identified, which are associated with light response, auxin response, gibberellin response, and methyl jasmonate response. Given that *P. notoginseng* is a shade-loving plant, the analysis focused on cis-acting elements related to light response, auxin response, and gibberellin response (Figure 6).

Among the identified elements, the light response-related cis-acting elements include Sp1, GT1-motif, 3-AF1 binding site, and AAAC-motif, which are distributed across 61 members of the *NAC* gene family. The gibberellin response-related cis-acting elements include P-box and GARE-motif, found in 33 *NAC* gene family members. The auxin-related cis-acting element is the TGA-element, present in 31 *NAC* gene family members. These results suggest that the *NAC* gene family in *P. notoginseng* may play a significant role in regulating the plant’s responses to light and hormonal stress.

### 3.6. Expression Pattern Analysis of the P. notoginseng NAC Gene Family

**Figure 7:**
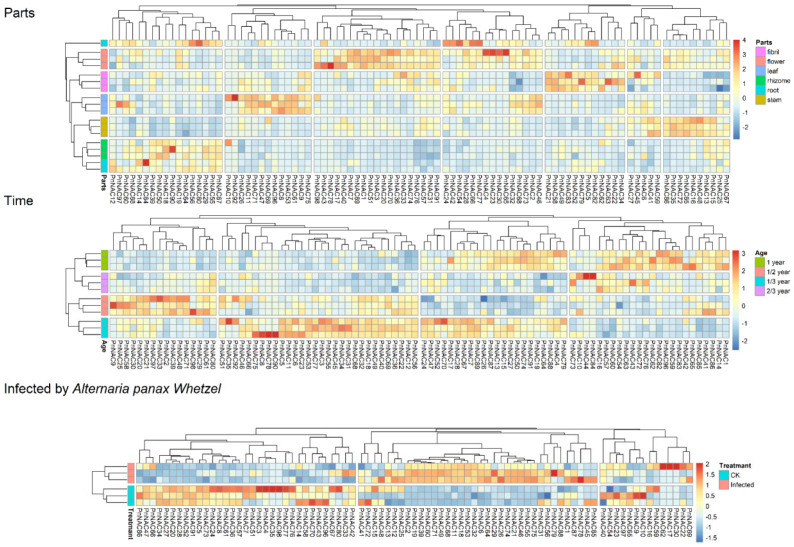
Expression patterns of the *P. notoginseng NAC* gene family members at different sites, stages, and in response to *A. panax* stress.

RNA-Seq analysis was conducted to investigate the expression patterns of the 98 members of the *NAC* gene family in *P. notoginseng* across different tissues, growth stages, and under stress from the black spot disease (Figure 7). Overall, the expression of NAC genes in *P. notoginseng* exhibits tissue specificity, with the highest expression levels found in the stems, leaves, and flowers, while a cluster of genes in the roots also shows notable expression specificity. The expression patterns of the 98 *NAC* gene family members vary at different time points, with the highest expression observed during the 1/3-year period.

These results indicate that the expression of the *NAC* gene family in *P. notoginseng* displays significant spatiotemporal specificity, suggesting that these genes play crucial roles throughout the plant’s growth process. Following infection by the pathogen causing black spot disease, the expression of *NAC* gene family members in *P. notoginseng* undergoes significant changes, highlighting the important role of these genes in the plant’s response to pathogenic invasion.

## 4. Discussion

NAC transcription factors are one of the largest gene families in plants, playing important roles in regulating plant growth and development, responding to environmental (biotic) stresses, and hormone signal transduction [12,29,30]. Studies have shown that the number of NAC transcription factors varies across different species. Ooka et al. [31] identified 105 and 75 NAC transcription factor genes in Arabidopsis and rice, respectively; Chen et al. [32] identified 114 NAC transcription factor genes in birch; Diao et al. [33] found 104 NAC transcription factor genes in pepper; Singh et al. [34] identified 110 NAC transcription factor genes in potato; and Liu et al. [18] found 89 NAC transcription factor genes in the closely related species ginseng. However, the structural characteristics and functions of the NAC transcription factor gene family in *P. notoginseng* remain unclear. Therefore, this study comprehensively analyzed the physicochemical properties, structural characteristics, and response patterns of the NAC transcription factor gene family in *P. notoginseng* under infection by black spot disease at different growth stages.

A total of 98 *PnNAC* family members with conserved NAC transcription factor domains were identified, which are similar to the number of NAC transcription factor genes in ginseng but lower than that in most other plants where NAC transcription factor gene family identification has been completed. This indicates that NAC transcription factor gene families are widely present in plants and that plant evolution may influence the distribution of these gene families. No tandem repeats were found among the members of the NAC transcription factor gene family, which is consistent with Liu et al. [18], who also did not observe tandem repeats among the NAC transcription factor members in ginseng. The absence of tandem repeats may be a potential reason for the significantly lower number of NAC genes in *P. notoginseng* and ginseng compared to other plants.

Cis-acting elements in the promoter regions of genes influence plant growth, development, and stress responses. The promoter regions of the NAC transcription factor gene family in *P. notoginseng* are rich in cis-acting elements related to hormone responses and light responses. As *P. notoginseng* is a shade-loving plant [35,36], its response to light is closely related to its growth, development, and stress responses. The light response-related cis-acting elements in the promoter regions of the *NAC* gene family members may play important roles in the plant’s responses to varying light conditions, warranting further investigation.

In summary, this study utilized transcriptomic data to find that the expression patterns of the NAC transcription factor gene family exhibit distinct characteristics at different growth stages and in various tissues of *P. notoginseng*. Following infection by the black spot disease pathogen (*A. panax* Whetzel), the expression of *PnNAC* genes showed significant changes. These results indicate that *PnNAC* plays an important role in the growth and development of *P. notoginseng* as well as in its response to biotic stresses. Due to the growth characteristics of *P. notoginseng*, this study did not use qPCR to validate the expression of relevant *PnNAC* genes. Future research should employ qPCR to further validate these expression patterns and delve deeper into the functional studies of *PnNAC*.

## Figures and Tables

**Table 1:** Physicochemical properties of the *NAC* gene family of *P. notoginseng*.

	Max	Median	Mean	Max
Length (AA)	104.00	344.00	357.00	882.00
Molecular weight (kDa)	11.78	38.73	40.70	100.20
IS	4.12	6.52	6.54	9.75

## Data Availability

The data presented in this study are available in the article.

## References

[1] Briskin D.P. (2000). Medicinal plants and phytomedicines. Linking plant biochemistry and physiology to human health. Plant Physiol..

[2] Ng T.B. (2006). Pharmacological activity of sanchi ginseng (*Panax notoginseng*). J. Pharm. Pharmacol..

[3] Mo Y., He X., Cui H., Cheng Y., Zhou M., Cui X., Zhang T. (2025). Gut microbiota: A new key of understanding for *Panax notoginseng* against multiple disorders and biotransformation. J. Ethnopharmacol..

[4] Guo D., Yu M., Guo H., Zeng M., Shao Y., Deng W., Qin Q., Li Y., Zhang S. (2024). *Panax notoginseng* saponins inhibits oxidative stress-induced human nucleus pulposus cell apoptosis and delays disc degeneration in vivo and in vitro. J. Ethnopharmacol..

[5] Pan Y.W., Wu D.P., Liang H.F., Tang G.Y., Fan C.L., Shi L., Ye W.C., Li M.M. (2022). Total saponins of *Panax notoginseng* activate Akt/mTOR pathway and exhibit neuroprotection in vitro and in vivo against ischemic damage. Chin. J. Integr. Med..

[6] Liu Y., Li S., Pu M., Qin H., Wang H., Zhao Y., Chen T. (2022). Structural characterization of polysaccharides isolated from *Panax notoginseng* medicinal residue and its protective effect on myelosuppression induced by cyclophosphamide. Chem. Biodivers..

[7] Mao Z.S., Wei F.G., Chen Z.J., Yang J.Y., Wang B.Y., Wang Y. (2017). Field investigation of round spot on sanqi (*Panax notoginseng*) in Yunnan province. J. Wenshan Univ..

[8] He C., Wang H.L., Jin X., Jin B.H., Su S., Duan Y.N., He X.H. (2020). Identification of *Alternaria* species acccosiated with black spot disease on *Panax notoginseng* in Yunnan and Guangxi. Plant Pathol..

[9] Chen K.K., Weng F., Qiang Y. (2014). Control effect of two kinds of medicament on powdery mildew of sedum aizoon linn. J. Shaanxi Agric. Sci..

[10] Li Y.B., Liu Y.X., Zhu S.S., Luo L.X., Li J.Q. (2020). Grading system for *Panax notoginseng* root rot disease. Plant Pathol..

[11] Yang L.F., Yang J., Gao L.L., Guo J.W., Hong L., Cheng J.X., Kong Q., Tian X.J. (2016). Screening and preliminary identification of soil bacteria antagonistic to *phytophthora* of *Panax notoginseng*. J. Honghe Univ..

[12] Shao H.B., Wang H.Y., Tang X.L. (2015). NAC transcription factors in plant multiple abiotic stress responses: Progress and prospects. Front. Plant Sci..

[13] Qu Y.T., Duan M., Zhang Z.Q., Dong J.L., Wang T. (2016). Overexpression of the *Medicago falcata* NAC transcription factor *MfNAC3* enhances cold tolerance in *Medicago truncatula*. Environ. Exp. Bot..

[14] Evans O. (2016). Functional Analysis of GhNAC18 and GhNAC20 Genes in Upland Cotton Leaf Senescence and Stress Response.

[15] Mao X.G., Chen S.S., Li A., Zhai C.C., Jing R.L. (2014). Novel NAC transcription factor TaNAC67 confers enhanced multi-abiotic stress tolerances in *Arabidopsis*. PLoS ONE.

[16] Chen N., Wu S.H., Fu J.L., Cao B.H., Lei J.J., Chen C.M., Jiang J. (2016). Overexpression of the eggplant (*Solanum melongena*) NAC family transcription factor *SmNAC* suppresses resistance to bacterial wilt. Sci. Rep..

[17] Shen J.B., LV B., Luo L.Q., He J.M., Mao C.J., Xi D.D., Ming F. (2017). The NAC-type transcription factor *OsNAC2* regulates ABA-dependent genes and abiotic stress tolerance in rice. Sci. Rep..

[18] Liu Q., Sun C.Y., Han J.Z., Li L., Wang K.Y., Wang Y.F., Chen J., Zhao M.Z., Wang Y., Zhang M.P. (2020). Identification, characterization and functional differentiation of the *NAC* gene family and its roles in response to cold stress in ginseng, *Panax ginseng* C.A. Meyer. PLoS ONE.

[19] Yang Z.J., Liu G.Z., Zhang G.H., Yan J., Dong Y., Lu Y., Fan W., Hao B., Lin Y., Li Y. (2021). The chromosome-scale high-quality genome assembly of *Panax notoginseng* provides insight into dencichine biosynthesis. Plant Biotechnol. J..

[20] Mistry J., Chuguransky S., Williams L., Qureshi M., Salazar G.A., Sonnhammer E.L.L., Tosatto S.C.E., Paladin L., Raj S., Richardson L.J. (2021). Pfam: The protein families database in 2021. Nucleic Acids Res..

[21] Finn R.D., Clements J., Eddy S.R. (2011). Hmmer web server: Interactive sequence similarity searching. Nucleic Acids Res..

[22] Sievers F., Wilm A., Dineen D., Gibson T.J., Karplus K., Li W.Z., Lopez R., McWilliam H., Remmert M., Soding J. (2011). Fast, scalable generation of high-quality protein multiple sequence alignments using Clustal Omega. Mol. Syst. Biol..

[23] Price M.N., Dehal P.S., Arkin A.P. (2010). FastTree 2—Approximately maximum-likelihood trees for large alignments. PLoS ONE.

[24] Yu G.C., David K.S., Zhu H.C., Guan Y., Tommy T.-Y.L. (2017). Ggtree: An R package for visualization and annotation of phylogenetic trees with their covariates and other associated data. Methods Ecol. Evol..

[25] Lescot M., Déhais P., Thijs G., Marchal K., Moreau Y., Peer Y.V., Rouzé P., Rombauts S. (2002). PlantCARE, a database of plant *cis*-acting regulatory elements and a portal to tools for in silico analysis of promoter sequences. Nucleic Acids Res..

[26] Bailey T.L., Nadya W., Chris M., Li W.W. (2006). MEME: Discovering and analyzing DNA and protein sequence motifs. Nucleic Acids Res..

[27] Kim D., Paggi J.M., Park C., Bennett C., Salzberg S.L. (2019). Graph-based genome alignment and genotyping with HISAT2 and HISAT-genotype. Nat. Biotechnol..

[28] Simon A., Theodor P.P., Wolfgang H. (2015). HTSeq—A Python framework to work with high-throughput sequencing data. Bioimformatics.

[29] Puranik S., Sahu P.P., Srivastava P.S., Prasad M. (2012). NAC proteins: Regulation and role in stress tolerance. Trends Plant Sci..

[30] Wang C.Y., Zhang Q. (2018). Research progress on the function of NAC transcription factors in plants. Biotechnol. Bull..

[31] Ooka H., Satoh K., Doi K., Nagata T., Otomo Y., Murakami K., Matsubara K., Osato N., Kawai J., Carninci P. (2003). Comprehensive analysis of NAC family genes in *Oryza sativa* and *Arabidopsis thaliana*. DNA Res..

[32] Chen S., Lin X., Zhang D., Li Q., Zhao X.Y., Su C. (2019). Genome-wide analysis of *NAC* gene family in *Betula pendula*. Forests.

[33] Diao W.P., Snyder J.C., Wang S.B., Liu J.B., Pan B.G., Guo G.J., Ge W., Dawood M.H.S.A. (2018). Genome-Wide Analyses of the NAC Transcription Factor Gene Family in Pepper (*Capsicum annuum* L.): Chromosome Location, Phylogeny, Structure, Expression Patterns, Cis-Elements in the Promoter, and Interaction Network. Int. J. Mol. Sci..

[34] Singh A.K., Sharma V., Pal A.K., Acharya V., Ahuja P.S. (2013). Genome-wide organization and expression profiling of the NAC transcription factor family in potato (*Solanum tuberosum* L.). DNA Res..

[35] Kuang S.B., Xu X.Z., Meng Z.G., Zhang G.H., Yang S.C., Chen Z.J., Wei F.G., Chen J.W. (2015). Effects of light transmittance on plant growth and root ginsenoside content of *Panax notoginseng*. Chin. J. Appl. Environ. Biol..

[36] Zhang J.Y., Zhang Q.H., Shang S.P., Cun Z., Wu H.M., Chen J.W. (2021). The Responses of light reaction of photosynthesis to dynamic sun flecks in a typically shade-tolerant species *Panax notoginseng*. Front. Plant Sci..

